# Novel role for PI3Kβ in placental function through regulation of system A amino acid transporter expression, associated with embryonic lethality

**DOI:** 10.1007/s00018-025-05937-w

**Published:** 2025-11-19

**Authors:** Sarah E. Conduit, Cindy X. W. Zhang, Wayne Pearce, Julie Guillermet-Guibert, Amanda N. Sferruzzi-Perri, Bart Vanhaesebroeck

**Affiliations:** 1 Cell Signalling, UCL Cancer Institute, University College London, London, UK; 2 Department of Physiology, Development, and Neuroscience, Loke Centre for Trophoblast Research, University of Cambridge, Cambridge, UK; 3 Labex Toucan, Toulouse, France; 4 Université de Toulouse, Inserm, CNRS, CRCT, Centre de Recherches en Cancérologie de Toulouse, Toulouse, France

**Keywords:** PI 3-kinase, *PIK3CB*, Placenta, Nutrient transporters, Drug target, Inhibitor

## Abstract

The placenta is essential for embryonic development, in part by mediating nutrient transfer from mother to embryo. Placental insufficiency is the most common cause of intrauterine growth restriction which has long-term health consequences lasting into adulthood. p110β is a class IA phosphoinositide 3-kinase (PI3K) catalytic subunit, a family of lipid kinases which are critical regulators of adult metabolism, immunity and embryonic and placental development. However, unlike the other class IA PI3K isoforms, the in vivo functions of p110β remain unclear. While homozygous p110β kinase-dead mice are mostly embryonically lethal, some survive into adulthood with no apparent phenotypes, other than reduced fertility. The mechanism(s) underlying this embryonic lethality remain unclear. Therefore, we performed an in-depth characterisation of p110β kinase-dead embryos, revealing a previously unrecognised role for p110β in controlling the expression of system A amino acid transporters. We show that homozygous p110β kinase-dead embryos are phenotypically normal, but growth-restricted and exhibit placental insufficiency. The placenta is small with a reduced nutrient storing junctional zone and downregulation of the system A amino acid transporters, required for maternal-to-embryo amino acid transfer. These data suggest defective amino acid transfer drives embryonic growth restriction and partial lethality of p110β kinase-dead embryos. This predominantly embryonic p110β phenotype is consistent with the notion that system A amino acid transporters are more critical during development than in adult physiology. The greater significance of p110β in development than in adult homeostasis may also help explain why p110β inhibitors, compared to inhibitors of other PI3K isoforms, are well-tolerated in adults.

**Supplementary Information:**

The online version contains supplementary material available at 10.1007/s00018-025-05937-w.

## Introduction

A balanced allocation of maternal nutrients to the embryo during development is essential for long-term health outcomes, with both intrauterine growth restriction (the inability of a fetus to attain its genetically defined growth potential), as well as overgrowth being associated with metabolic disease later in life [1–3]. Nutrient allocation and balancing are achieved by the placenta which responds to the maternal availability of nutrients and embryonic demands. Although the placenta has a large dynamic range to balance the competing maternal and embryonic needs and extensive compensatory mechanisms which evolve over pregnancy [4], placental insufficiency is the most common cause of intrauterine growth restriction in the developed world [5].

The murine placenta consists of the embryo-derived labyrinthine and junctional zones and the maternal endometrium-derived decidua. The labyrinthine zone contains maternal and embryonic blood spaces and is the site of oxygen and nutrient transfer to the embryo. The junctional zone sits between the labyrinthine zone and decidua, producing hormones and storing energy. The decidua aids in immune protection of the embryo and provides structural and nutritional support prior to full placental development.

The class IA phosphoinositide 3-kinases (PI3Ks) are heterodimeric lipid kinases consisting of a p110 catalytic subunit (p110α, p110β, p110δ encoded by *PIK3CA*, *PIK3CB* and *PIK3CD*, respectively) and a p85 regulatory subunit which inhibits p110 activity in the basal state and recruits the complex to activated receptors following stimulation [6]. These PI3Ks play critical roles during development and in adults, which is underscored by the wide-ranging phenotypic consequences of deletion or kinase inactivation of the ubiquitously-expressed p110α and p110β or the immune-cell enriched p110δ in mice (reviewed in Refs [6, 7]). Activated class I PI3Ks phosphorylate the membrane lipid PI(4,5)P_2_, producing PI(3,4,5)P_3_ (also known as PIP_3_) leading to the recruitment, phosphorylation and activation of the serine/threonine kinases AKT1, AKT2 and AKT3, which modulate the activity of numerous downstream effectors including mTORC1 (reviewed in Ref [6]).

Genetic manipulation of the PI3K pathway in the highly sensitive context of embryonic development has revealed conserved cell biological functions of PI3K pathway components of relevance to both development, as well as adult homeostasis and disease [8–14]. Mouse models with a germline kinase-inactivating point mutation have been particularly instrumental to characterise the in vivo catalytic functions played by these kinases, without impacting kinase-independent scaffolding functions and disrupting PI3K-family stoichiometry [8, 15–17].

PI3Kα is key in metabolic signalling and plays essential roles downstream of the insulin and insulin like growth factor-1 (IGF-1) receptors [16]. Ubiquitous homozygous germline constitutive kinase inactivation of p110α in *Pik3ca*^*D933A/D933A*^ mice leads to growth-retardation from embryonic day 9 (E9) onwards and embryonic lethality around E10.5, with defective angiogenesis as a prominent phenotype [8].

Adult heterozygous *Pik3ca*^*+/D933A*^ mice are viable and fertile [16, 18]. Interestingly however, these mice (*Pik3ca*^*+/D933A*^) exhibit embryonic growth restriction with disrupted placental development [16, 18]. The *Pik3ca*^*+/D933A*^ placenta is small and the labyrinthine zone, including the surface area for nutrient exchange, is reduced [18]. After birth, *Pik3ca*^*+/D933A*^ mice remain smaller than littermate controls up to 6 months of age due to a reduction in mass, specifically in skeletal muscle [16]. Pregnant *Pik3ca*^*+/D933A*^ dams exhibit increased placental mass and junctional zone area, but reduced glucose transport to embryos, regardless of embryo genotype [18]. Therefore, while loss of p110α kinase activity in the pregnant mother increases placental mass and likely nutrient storage, its loss in the embryo/placenta reduces placental mass and nutrient transfer [18]. AKT1 appears to be the key AKT isoform in the placenta [19]. Ubiquitous deletion of *Akt1* induces intrauterine growth restriction with low placental mass, reduced glycogen cells, disorganised and fewer blood vessels and a disproportional loss of the decidua [19, 20]. Furthermore, AKT and mTORC1 signalling are downregulated in rat and non-human primate models of low protein diet-induced intrauterine growth restriction [21, 22].

In contrast to p110α and p110δ, which are key in organismal metabolic regulation and immunity respectively, the biological functions of the ubiquitously expressed p110β remain less clear. p110β integrates signalling by coincident detection of activated receptor tyrosine kinases (RTKs), G protein-coupled receptors (GPCRs) and small GTPases (CDC42, RAC1 and RAB5) [23–25]. Mice with germline global deletion of p110β (*Pik3cb*^*−/−*^) exhibit embryonic lethality at E3.5 [26]. In contrast, germline ubiquitous kinase inactivation of p110β in mice (*Pik3cb*^*D931A/D931A*^ and *Pik3cb*^*K805R/K805R*^) is only partially embryonically lethal via unknown molecular mechanisms [27, 28]. In *Pik3cb*^*D931A/D931A*^ mice, waves of lethality are observed at E8.5–10.5 and E14.5-E16.5 on a C57BL/6 × 129S2/Sv or C57BL/6 × 129S2/Sv x BALB/c mixed genetic background, with a slightly more severe phenotype on a C57BL/6 background [27]. Given the differences observed between *Pik3cb* gene knock-out and knock-in approaches, p110β scaffolding functions likely contribute to the early embryonic lethality of null embryos, with PI3Kβ kinase activity likely being more important in specific cell types and contexts. Indeed, surviving p110β kinase-dead mice are generally healthy, other than mild insulin resistance beyond 6 months, subfertility in females and complete infertility in males [27–29]. This finding may help to explain the observation that PI3Kβ inhibitors are very well-tolerated in humans [30–32], compared to PI3Kα inhibitors.

At present, the role PI3Kβ plays in embryonic development and placental function and the mechanisms of lethality in p110β kinase-dead embryos remain unknown. Interestingly, p110β expression is downregulated in the placenta of undernourished pregnant mice prior to the onset of fetal growth restriction [33]. Here we investigated the phenotype and underlying molecular mechanisms of PI3Kβ kinase-dead embryos (using the *Pik3cb*^*D931A/D931A*^ mouse). We show the majority of E10.5 and E13.5 *Pik3cb*^*D931A/D931A*^ embryos are phenotypically normal, but growth-restricted compared to littermate controls and exhibit placental insufficiency. The *Pik3cb*^*D931A/D931A*^ placenta exhibits structural and functional deficits including reduced expression of system A amino acid transporters, which are essential for amino acid transfer to the embryo and for embryonic growth.

## Materials and methods

### Antibodies and reagents

Antibodies used were: GAPDH (ab8245, IB 1:10,000) was from Abcam (Cambridge, MA, USA). pAKT(S473) (9271, IB 1:1,000), pAKT(T308) (4056, IB 1:500), AKT (9272, IB 1:1,000), p110α (4249, IB 1:1,000), p110β (3011, IB 1:1,000), pS6RP(S240/244) (2215, IB 1:1,000) and S6RP (2317, IB 1:1,000) were from Cell Signaling Technology (Danvers, MA, USA). Secondary antibodies were from Cell Signaling Technology; Anti-rabbit IgG, HRP-linked Antibody (7074, IB 1:3,000), Anti-mouse IgG, HRP-linked Antibody (7076, IB 1:3,000).

### Mouse studies

*Pik3cb*^*tm2.1Bvan*^ (hereafter, *Pik3cb*^*D931A*^) mice were generated and described previously [27]. Mice were backcrossed onto the C57BL/6 background for at least 6–10 generations. Mice were maintained in individually-ventilated cages in a specific pathogen-free facility with a 07:30 − 19:30 light and 19:30 − 07:30 dark cycle at 18–22 °C and 40–60% humidity. Embryos age is indicated in the relevant figure legends. Male and female embryos were analysed separately and combined as indicated in the figures and legends.

Yolk sacs or embryo tissue were used for genotyping of embryos and ear notches were used for genotyping of postnatal mice via PCR analysis of genomic DNA using the following primers: for *Pik3cb* b11: CTTAGGGAAGAGCGAGGA, Bseq2: AAGAAGTATGTACACCTCTCT, Neo F2: CTGTCATCTCACCTTGCTCC. Sex genotyping was performed as described previously [34] using embryonic tissue and the following primers: *Jarid1c* and *Jarid1d* forward: CTGAAGCTTTTGGCTTTGAG, *Jarid1c* and *Jarid1d* reverse: CCACTGCCAAATTCTTTGG.

Stereo microscopy of embryos and placentas was performed using a Leica M205FA stereo microscope with Leica Application Suite acquisition software. Crown rump length was measured in bright field embryo images using Fiji(ImageJ).

### Histology

Mouse placentas were bisected in the midline and formalin-fixed, paraffin-embedded (FFPE) sections prepared and stained with haematoxylin and eosin (H&E). H&E-stained placenta sections were imaged using a Hamamatsu NanoZoomer S360 slide scanner with 20x or 40x objectives. The percentage areas of placental labyrinthine zone and junctional zone and junctional zone cell type proportions were assessed by point counting using Fiji ImageJ software (National Institutes of Health, USA) as described previously [18].

### Mouse embryonic fibroblasts (MEFs) for Immunoblotting

MEFs were generated from E13.5 embryos as described previously [35] and cultured in DMEM containing 10% FBS and 1% penicillin-streptomycin at 37 °C and 5% CO_2_. MEFs were serum starved overnight and stimulated with 20 ng/ml SDF-1 (Peprotech, Cranbury, NJ, USA, 250–20 A), 1 µM LPA (Biomol, Hamburg, Germany), 20 ng/ml IL8 (Peprotech), PDGF-BB (Peprotech, 100-14B), 1 µM insulin (Peprotech), 25 ng/ml IGF-1 (Peprotech, 250 − 19) for 10 min prior to lysis.

### Immunoblot analysis

For mouse placenta immunoblots, approximately 100 mg of snap frozen mouse placental tissue was thawed in 500 µl RIPA buffer (50 mM HEPES pH 7.4, 150 mM NaCl, 10% glycerol, 1.5 mM MgCl_2_, 1 mM EGTA, 1 mM Na_3_VO_4_, 1% volume/volume (v/v) Triton X-100, 1% weight/volume (w/v) sodium deoxycholate, 0.1% w/v sodium dodecyl sulphate (SDS), 100 mM NaF, with phosphatase (524625-1SET; Merck, Rahway, NJ, USA) and protease (539131-10Vl; Merck) inhibitors) on ice. Tissue was homogenised in 2 ml Lysing Matrix tubes (MP-Biomedicals, Santa Ana, CA, USA) with beads (1/4 inch ceramic beads) using a FastPrep 24 homogeniser (MP Biomedicals) at 4 m/sec for 20 s. To remove debris, samples were transferred to fresh tubes and centrifuged at 20,800 g for 20 min at 4 °C, transferred to a second tube, followed by a second centrifugation step 20,800 g for 10 min at 4 °C.

MEFs lysates were harvested in lysis buffer (50 mM Tris.HCl pH 7.5, 150 mM NaCl, 1 mM EDTA, 1% v/v Triton X-100, Supplemented with Sodium orthovanadate, DTT 1mM, NaF 2mM, cOmplete Mini protease inhibitor (Roche)) for 10 min on a wheel at 4 °C and then centrifuged at 13,000 rpm for 10 min.

Protein concentration of the supernatant was determined using the Bradford assay (500-0006, BioRad, Hercules, CA, USA). Proteins were resolved using SDS-PAGE using NuPAGE 4–12% Bis-Tris Gel, 1 mm gels (ThermoFisher Scientific, Waltham, MA, United States), transferred to nitrocellulose membranes (IB23001 × 3; ThermoFisher Scientific) using dry transfer (iBlot2, ThermoFisher Scientific). 5% BSA was used for blocking, membranes were then incubated with primary antibodies overnight at 4 °C followed by secondary antibodies at room temperature for 1 h before detection by chemiluminescence (WBLUF0500, Immobilon Forte Western HRP substrate, Merck) using a Gel Doc (Bio-Rad). Where required, membranes were stripped using Guanidine Hydrochloride pH 3 (Sigma, G4505) prior to re-probing.

### MEFs for quantitative reverse transcription PCR

Immortalised WT MEFs were generated as described previously [36] and cultured in DMEM containing 10% FBS and 1% penicillin-streptomycin at 37 °C and 5% CO_2_. MEFs were seeded and cultured overnight prior to RNA extraction.

### Quantitative reverse transcription PCR

RNA was isolated from placentas or cells using the Direct-Zol RNA/DNA kit (Zymo Research, Irvine, CA, USA, R2080). cDNA synthesis was performed using 90 ng of RNA using the High-Capacity cDNA Reverse Transcription Kit (Thermo Fisher Scientific, Waltham, MA, USA, 4368814) according to the manufacturer instructions. cDNA samples were diluted 1:10 and analysed by qPCR using PowerUp SYBR Green Master Mix for qPCR (Thermo Fisher Scientific, A25742) and QuantiTect Mm_*Slc2a1*_1_SG, Mm_*Ctsq*_1_SG, Mm_*Syna*_1_SG, Mm_*Gcm1*_1_SG, Mm_*Prl3d1*_1_SG, Mm_*Prl8a8*_1_SG, Mm_*Prl3b1*_1_SG, Mm_*Hand1*_1_SG, Mm_*Mest*_1_SG, Mm_*Slc2a3*_1_SG, Mm_*Slc38a1*_1_SG, Mm_*Slc38a2*_1_SG, Mm_*Slc38a4*_1_SG and Mm_*Actb*_1_SG (249900, Qiagen, Hilden, Germany) primers with a QuantStudio 5 Real-Time PCR System or QuantStudio 6 Real-Time PCR System. All samples were analysed in duplicate and expression normalized to *Actb* which was unchanged between genotypes using the ΔΔCt method [37].

### Single nuclei RNAseq analysis

Single nuclei RNAseq analysis of E9.5, E10.5, E12.5 and E14.5 mouse placenta was performed as described previously [38]. R objects of process data were analysed in RStudio (2023.09.01 + 494) using R (4.4.3) and the Seurat package (5.2.1). The R objects AllStages_AllNuclei_obj.Rdata and AllStages_TrophoblastNuclei_obj.Rdata were downloaded from https://figshare.com/projects/Single_nuclei_RNA-seq_of_mouse_placental_labyrinth_development/92354 on the 09/04/2025 and 16/04/2025, respectively. Dot plots were generated for *Pik3ca*, *Pik3cb*, *Pik3cg*, *Pik3cd*, *Slc38a1*, *Slc38a2* and *Slc38a4* using the RNA assay and the DimPlot function.

### Statistical analysis

Statistical analysis was performed using GraphPad Prism 10.1.2 or InVivoStat v4.10.0 with graphs representing mean ± SD. Differences between groups were considered statistically significant when *p* < 0.05. Relevant statistical tests and sample sizes are indicated in the appropriate figure legends. Unpaired, two-sided tests were used. For Student’s t tests, sample variance was assessed by the F test to determine whether to use Welch’s correction for unequal variance. For single measures parametric analysis, mouse litter was used as a block factor to control for differences between embryos and placentas from different litters. Sample sizes were chosen based on our experience and standards in the field. No data were excluded from the analyses. Mouse embryos were randomised based on their genotype and sex and investigators were blind to embryo genotype during analysis.

## Results

### PI3Kβ kinase inactivation is partially embryonically lethal with growth-restricted surviving embryos

**Fig. 1:**
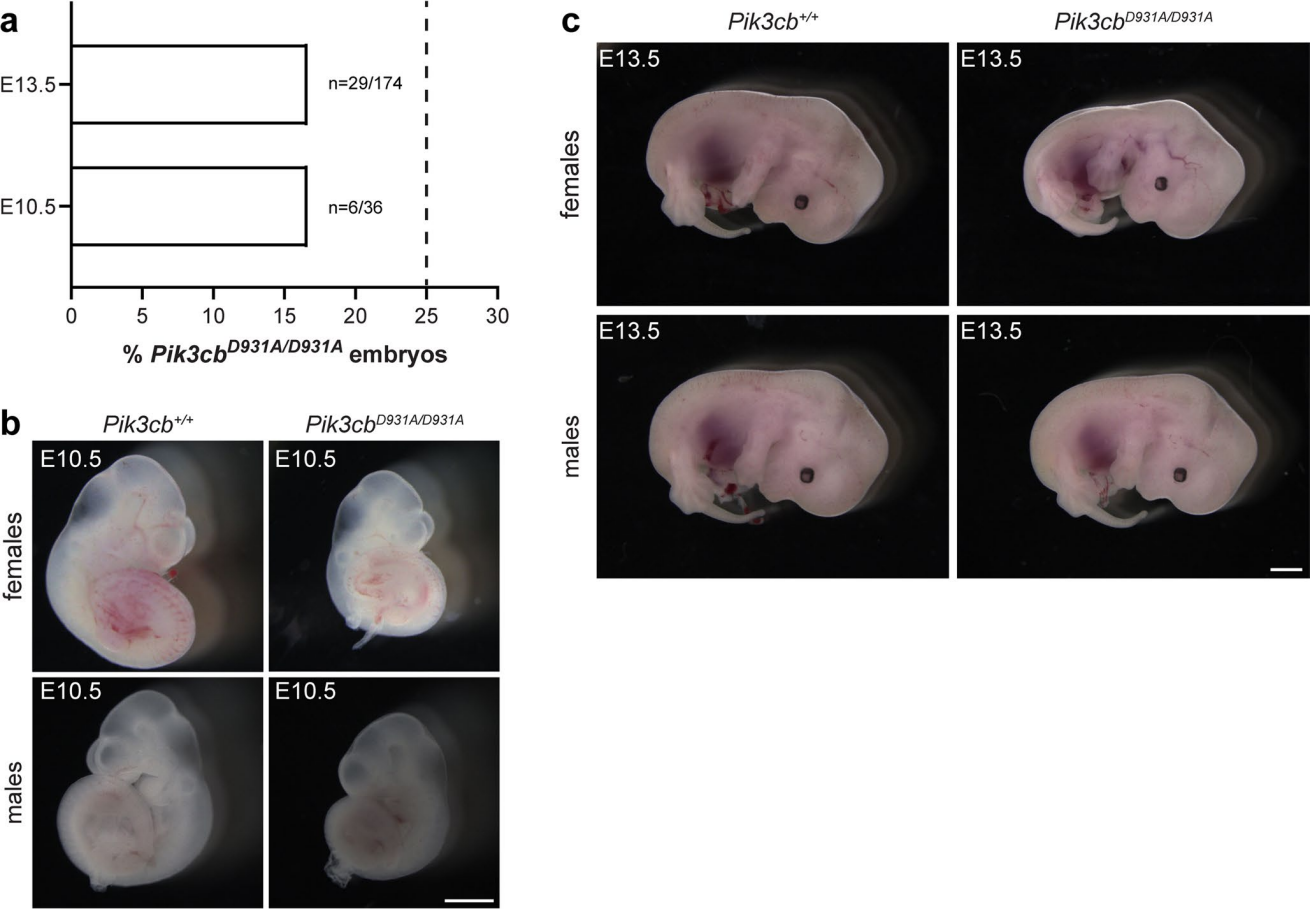
PI3Kβ inactivation is partially embryonically lethal **a.** Percentage of *Pik3cb*^*D931A/D931A*^ embryos observed from *Pik3cb*^*D931A/+*^ inter-crosses at indicated gestational days, dotted line indicates expected Mendelian ratio. E10.5 *n* = 6 *Pik3cb*^*D931A/D931A*^ embryos from 36 embryos from 4 litters, E13.5 *n* = 29 *Pik3cb*^*D931A/D931A*^ embryos from 174 embryos from 19 litters **b-c.** Whole mount images of (**b**) E10.5 or (**c**) E13.5 *Pik3cb*^*+/+*^ and *Pik3cb*^*D931A/D931A*^ embryos, scale bar: (**b**) 500 μm (**c**) 1 mm, representative of (**b**) *n* = 14 (*Pik3cb*^*+/+*^), *n* = 6 (*Pik3cb*^*D931A/D931A*^), (**c**) *n* = 38 (*Pik3cb*^*+/+*^), *n* = 28 (*Pik3cb*^*D931A/D931A*^) embryos

To investigate the mechanism(s) underlying the *Pik3cb*^*D931A/D931A*^ embryonic phenotype, *Pik3cb*^*D931A/+*^ mice were inter-crossed and embryos analysed at E10.5 and E13.5 on a C57BL/6 background. At both time points, the percentage of surviving *Pik3cb*^*D931A/D931A*^ embryos was reduced to 16.7%, compared to the expected Mendelian ratio of 25.0% (Fig. 1a). The majority of male and female embryos were morphologically normal at E10.5 and E13.5 but smaller than their *Pik3cb*^*+/+*^ litter-mate controls (Fig. 1b, c). *Pik3cb*^*+/D931A*^ embryos were undistinguishable to *Pik3cb*^*+/+*^ (Fig. S1a). A small number (2 out of 28) of *Pik3cb*^*D931A/D931A*^ embryos exhibited more severe patterning defects including craniofacial abnormalities and anophthalmia at E13.5 (Fig. S1b), but due to the low frequency of this observation these phenotypes were not the focus of this study. No overt difference in sex ratio was observed in embryos of any genotype (Fig. S1c).

### Embryo-placental growth is reduced in embryos with inactive PI3Kβ

Growth restriction is often characterised by defects in IGF-1/Insulin-like Growth Factor 1 Receptor (IGF1R) signalling and placental insufficiency [39–41]. Given that class I PI3Ks signal downstream of RTKs such as the IGF1R, we first asked whether IGF-1 signalling is perturbed in *Pik3cb*^*D931A/D931A*^ cells.

*Pik3cb*^*D931A/D931A*^ mouse embryonic fibroblasts (MEFs) stimulated with IGF-1, or additional RTK ligands PDFG and insulin, exhibited similar phosphorylation and activation of the effector kinase AKT relative to wild-type (WT) MEFs (Fig. S1d). In contrast, the response to GPCR ligands SDF-1 and LPA was abrogated by PI3Kβ inactivation (Fig. S1d), indicating a predominant role for PI3Kβ downstream of GPCR rather than RTK ligands and suggesting repression of IGF-1 signalling is unlikely to play a role in *Pik3cb*^*D931A/D931A*^ embryonic growth restriction.

**Fig. 2:**
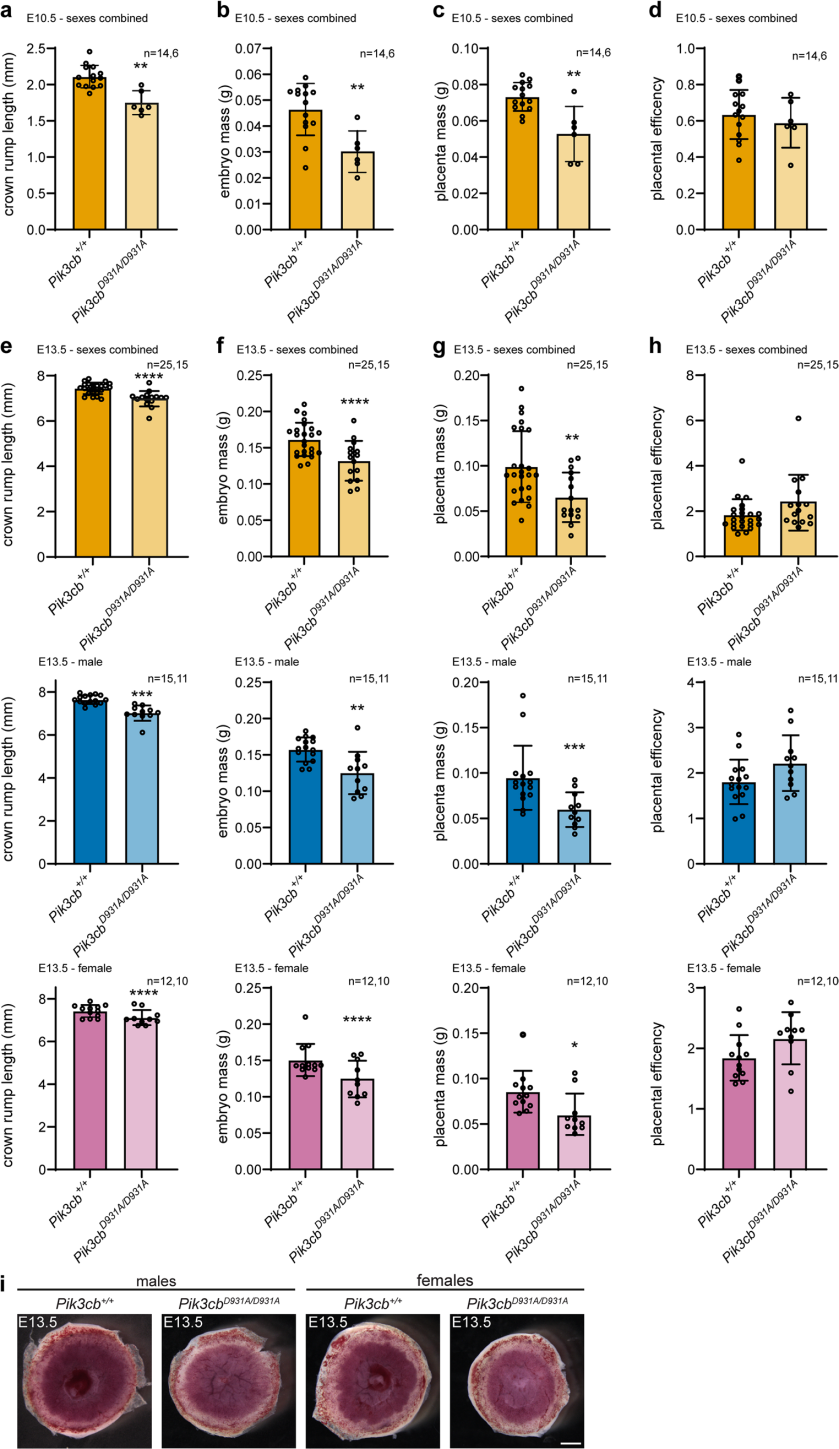
Reduced feto-placental growth in embryos with PI3Kβ inactivation **a-d.** E10.5 *Pik3cb*^*+/+*^ and *Pik3cb*^*D931A/D931A*^ (**a**) embryo crown-rump length, (**b**) embryo mass, (**c**) placental mass and (**d**) placental efficiency. Bars represent mean ± SD, *n* = 14 (*Pik3cb*^*+/+*^), *n* = 6 (*Pik3cb*^*D931A/D931A*^) embryos, **p < 0.01, (single measures parametric analysis, with litter as a block factor (**a**) *p* = 0.0014, (**b**) *p* = 0.0070, (**c**) *p* = 0.0067, (**d**) *p* = 0.6210) **e-h.** E13.5 *Pik3cb*^*+/+*^ and *Pik3cb*^*D931A/D931A*^ sexes combined, male and female (**e**) embryo crown-rump length, (**f**) embryo mass, (**g**) placental mass and (**h**) placental efficiency. Bars represent mean ± SD, *n* = 25 (sexes combined *Pik3cb*^*+/+*^), *n* = 15 (sexes combined *Pik3cb*^*D931A/D931A*^), *n* = 15 (male *Pik3cb*^*+/+*^), *n* = 11 (male *Pik3cb*^*D931A/D931A*^), *n* = 12 (female *Pik3cb*^*+/+*^), *n* = 10 (female *Pik3cb*^*D931A/D931A*^) embryos, *p < 0.05, **p < 0.01, ***p < 0.001, ****p < 0.0001 (single measures parametric analysis, with litter as a block factor, (**e**) combined *p* < 0.0001, male *p* = 0.0003, female *p* < 0.0001, (**f**) combined *p* < 0.0001, male *p* = 0.0080, female *p* < 0.0001, (**g**) combined *p* = 0.0013, male *p* = 0.0003, female *p* = 0.0207, (**h**) combined *p* = 0.3730, male *p* = 0.0663, female *p* = 0.2354) **i.** Whole mount images of *Pik3cb*^*+/+*^ and *Pik3cb*^*D931A/D931A*^ placentas, scale bar 1 mm, representative of *n* = 15 (male *Pik3cb*^*+/+*^), *n* = 11 (male *Pik3cb*^*D931A/D931A*^), *n* = 12 (female *Pik3cb*^*+/+*^), *n* = 10 (female *Pik3cb*^*D931A/D931A*^) embryos

Resource allocation to the developing embryos by the placenta is another critical determinant of embryo size [42]. We therefore investigated placental and embryo growth in *Pik3cb*^*D931A/D931A*^ embryos. First, we confirmed that *Pik3cb* mRNA is expressed in the WT mouse placenta (Fig. S2a). At E10.5, embryo crown rump length, embryo mass and placental mass were reduced in *Pik3cb*^*D931A/D931A*^ embryos compared to litter-mate *Pik3cb*^*+/+*^ controls (Fig. 2a, b, S2b, c). However, placental efficiency (measured by the ratio of embryo mass to placental mass) was unchanged at E10.5 (Fig. 2d, S2e). Similarly, at E13.5 crown rump length, embryo mass, placental mass, but not placental efficiency, were reduced with PI3Kβ inactivation in both male and female embryos (Fig. 2e-h). The average *Pik3cb*^*D931A/D931A*^ embryo growth restriction was 35% at E10.5 but only 19% at E13.5, whereas placental growth restriction was 28% and 33% at E10.5 and E13.5 respectively, suggesting potential activation of placental compensation mechanisms at the later developmental stage. Although smaller and slightly pale, *Pik3cb*^*D931A/D931A*^ gross placental morphology was unchanged compared to controls (Fig. 2i).

### PI3Kβ inactivation disrupts placental junctional zone linage development

**Fig. 3:**
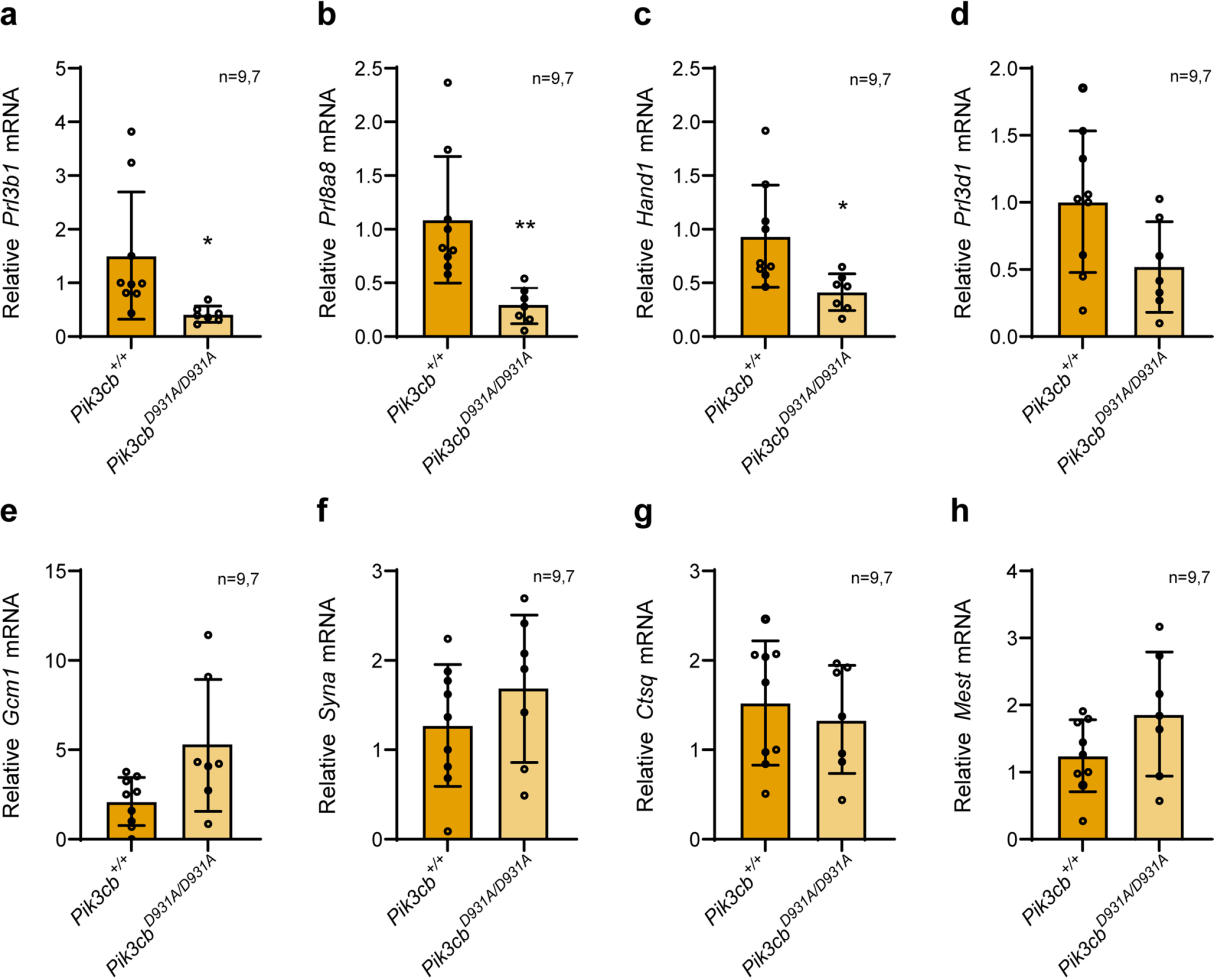
PI3Kβ inactivation reduces junctional zone linage markers **a-h.** E13.5 *Pik3cb*^*+/+*^ and *Pik3cb*^*D931A/D931A*^ placentas (sexes combined) were lysed and (**a**) *Prl3b1*, (**b**) *Prl8a8*, (**c**) *Hand1*, (**d**) *Prl3d1*, (**e**) *Gcm1*, (**f**) *Syna*, (**g**) *Ctsq*, (**h**) *Mest* mRNA levels quantified by qRT-PCR relative to *Actb*. Bars represent mean ± SD, *n* = 9 (*Pik3cb*^*+/+*^), *n* = 7 (*Pik3cb*^*D931A/D931A*^), *p < 0.05, **p < 0.01 (two-sided Student’s t-test (**a**) *p* = 0.0249, (**b**) *p* = 0.0033, (**c**) *p* = 0.0117, (**d**) *p* = 0.0527, (**e**) *p* = 0.0680, (**f**) *p* = 0.2943, (**g**) *p* = 0.5914, (**h**) *p* = 0.1129)

To begin to uncover the impact of PI3Kβ inactivation on placental development we assessed the expression of placental cell lineage markers [18] in E13.5 *Pik3cb*^*D931A/D931A*^ and control placentas via qRT-PCR. Notably, we observed a specific defect in the development of the placental energy storing and endocrine junctional zone, with reduced expression of junctional zone spongiotrophoblast markers *Prl3b1* (Fig. 3a) and *Prl8a8* (Fig. 3b) and giant cell marker *Hand1* (Fig. 3c), with a trend for reduced giant cell *Prl3d1* expression (Fig. 3d). Consistent trends were detected when embryos were categorized by sex, although with less significance, likely due to the lower power analysis following subgrouping (Fig. S3a-d).

Not significant differences between genotypes were observed in *Gcm1* (Fig. 3e, S3e) and *Syna* (Fig. 3f, S3f) which define the syncytiotrophoblasts in the nutrient exchanging labyrinthine zone, *Ctsq* (Fig. 3g, S3g) which defines the labyrinthine zone sinusoidal giant cells and in the embryonic endothelial cell marker *Mest* (Fig. 3h, S3h). Thus, PI3Kβ inactivation impairs junctional zone linage development, but has little impact on the specification of labyrinthine zone cell types.

### *Pik3cb*^*D931A/D931A* ^placentas exhibit a reduced junctional zone

**Fig. 4:**
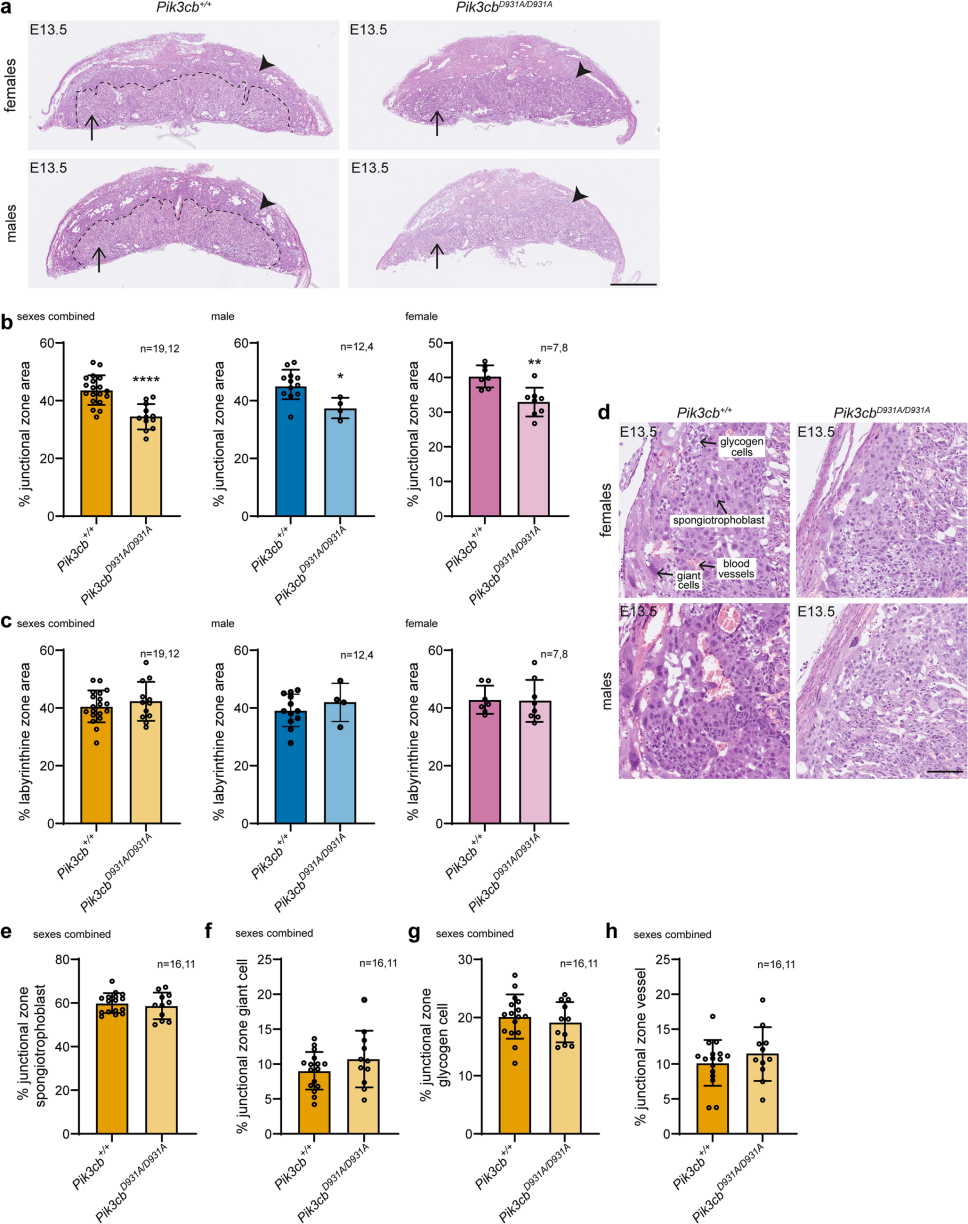
PI3Kβ inactivation reduces the placental junctional zone **a-c.** E13.5 *Pik3cb*^*+/+*^ and *Pik3cb*^*D931A/D931A*^ placenta sections were stained with H&E. (**a**) Arrow heads indicate junctional zone, arrows indicate labyrinthine zone, dotted line indicates the well-defined labyrinthine zone-junctional zone interface in the *Pik3cb*^*+/+*^ placenta, scale bar indicates 1 mm. The percentage of placental area occupied by the (**b**) junctional zone or (**c**) labyrinthine zone was quantified. Bars indicate mean ± SD, *n* = 19 (sexes combined *Pik3cb*^*+/+*^), *n* = 12 (sexes combined *Pik3cb*^*D931A/D931A*^), *n* = 12 (male *Pik3cb*^*+/+*^), *n* = 4 (male *Pik3cb*^*D931A/D931A*^), *n* = 7 (female *Pik3cb*^*+/+*^), *n* = 8 (female *Pik3cb*^*D931A/D931A*^) placentas of each genotype, *p < 0.05, **p < 0.01, ****p < 0.0001 (two-sided Student’s t-test (**b**) combined *p* = 1.653 × 10^−5^, male *p* = 0.0111, female *p* = 0.0021, (**c**) combined *p* = 0.4448, male *p* = 0.4349, female *p* = 0.8993) **d-h.** E13.5 *Pik3cb*^*+/+*^ and *Pik3cb*^*D931A/D931A*^ placenta sections were stained with H&E and the junctional zone is shown. Representative spongiotrophoblasts, giant cells, glycogen cells and blood vessels are indicated. (**d**) Scale bar indicates 100 μm. The percentage of the junctional zone occupied by the (**e**) spongiotrophoblast, (**f**) giant cells, (**g**) glycogen cells or (**h**) vessels was quantified. Bars indicate mean ± SD, *n* = 16 (sexes combined *Pik3cb*^*+/+*^), *n* = 11 (sexes combined *Pik3cb*^*D931A/D931A*^), placentas of each genotype (two-sided Student’s t-test (**e**) *p* = 0.5392, (**f**) *p* = 0.2081, (**g**) *p* = 0.5104, (**h**) *p* = 0.3648)

We next assessed whether the reduction in placental growth and expression of junctional zone linage markers (*Prl3b1* and *Prl8a8*) upon PI3Kβ inactivation were associated with histological changes in the placenta. We measured the percentage midline placental area occupied by the junctional and labyrinthine zones in E13.5 *Pik3cb*^*D931A/D931A*^ and *Pik3cb*^*+/+*^ placentas using point counting of the H&E-stained sections, a robust method of placental zone and cell type quantification [43]. In both male and female placentas, the percent junctional zone area was reduced upon PI3Kβ inactivation (Fig. 4a-b), consistent with the reduced expression of junctional zone linage markers. No changes in labyrinthine zone area were observed (Fig. 4a-c). The characteristic morphological distinction at the labyrinthine zone-junctional zone boundary was also less well defined in *Pik3cb*^*D931A/D931A*^ placentas (Fig. 4a). Despite a smaller junctional zone in *Pik3cb*^*D931A/D931A*^ placentas, the junctional zone area made up by of each composite cell type (spongiotrophoblasts, giant cells, glycogen cells and blood vessels) was unchanged when analysed as combined sexes and in females (Fig. 4d-h, S4a-d, a modest significant change in the spongiotrophoblast and giant cell area were observed specifically in the males Fig. S4a-b). These data suggest an overall defect in junctional zone development or maintenance, rather than the loss of a specific junctional zone cell type.

A small junctional zone with reduced spongiotrophoblasts, glycogen cells and giant cells, independent of changes in labyrinthine zone size, is observed in multiple murine models of embryo growth restriction and partial lethality, possibly via placental endocrine defects and thereby abnormal allocation of nutrients to the embryo [42, 44–46]. This suggests the small junctional zone in *Pik3cb*^*D931A/D931A*^ placentas may contribute to the small, but morphologically normal embryonic phenotype and partial lethality of these embryos.

### System A amino acid transporter expression is downregulated upon PI3Kβ inactivation

Placental efficiency is often increased in mammalian experimental models with small placentas as a compensatory mechanism that enhances the ability to transfer nutrients to the embryo. This occurs via histological changes in the labyrinthine zone, increasing the surface area for exchange and minimising barrier thickness, or by upregulating the expression of nutrient (amino acid and glucose) transporters [4, 47]. We observed no change in *Pik3cb*^*D931A/D931A*^ placental efficiency despite reduced placenta size. Therefore, compensation mechanisms that act to increase placental efficiency in small placentas may be defective in *Pik3cb*^*D931A/D931A*^ placentas and reduced transport of nutrients may contribute to embryo growth restriction.

**Fig. 5:**
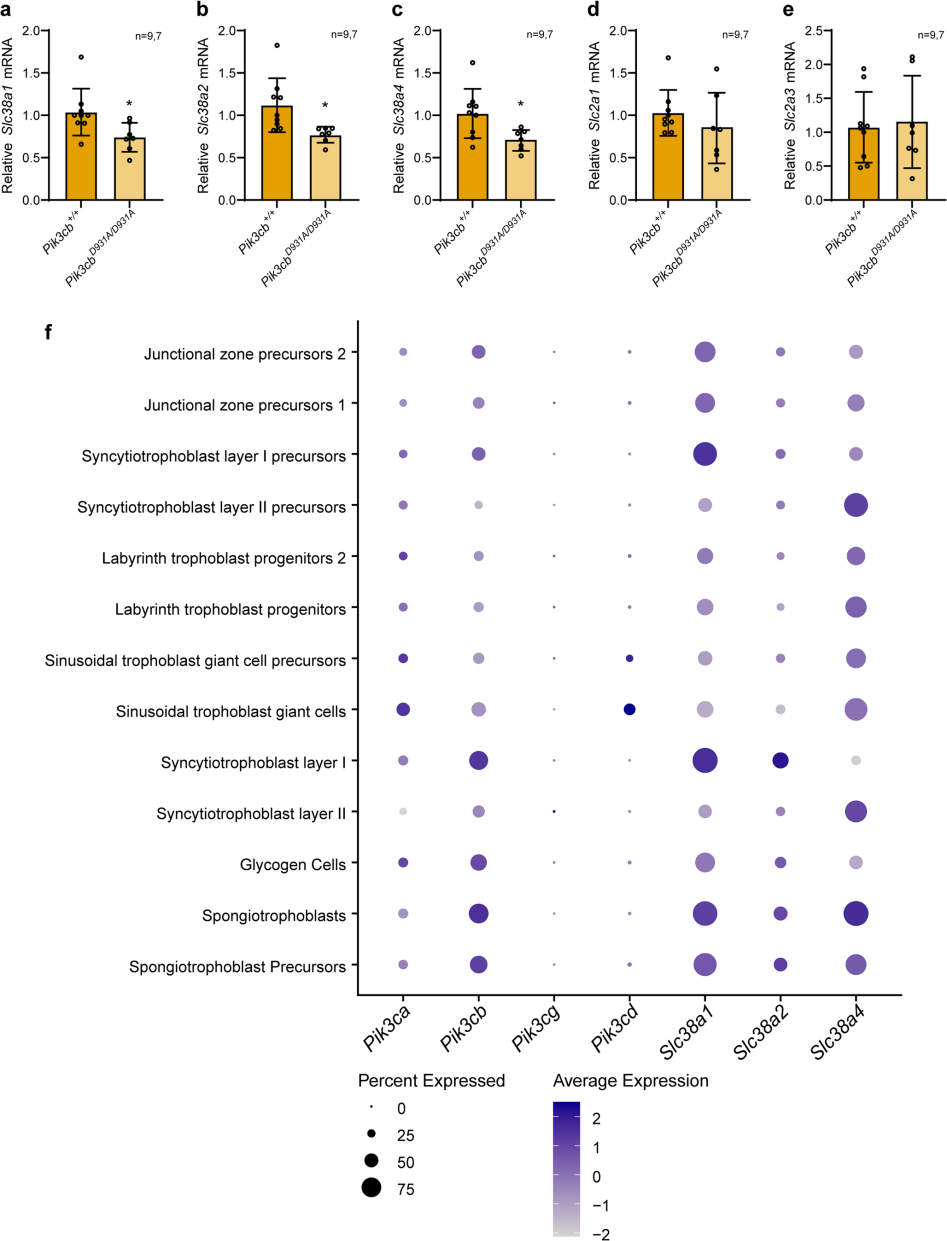
PI3Kβ inactivation reduces System A amino acid transporter expression **a-e.** E13.5 *Pik3cb*^*+/+*^ and *Pik3cb*^*D931A/D931A*^ placentas (sexes combined) were lysed and (**a**) *Slc38a1*, (**b**) *Slc38a2*, (**c**) *Slc38a4*, (**d**) *Slc2a1*, (**e**) *Slc2a3* mRNA levels quantified by qRT-PCR relative to *Actb*. Bars represent mean ± SD, *n* = 9 (*Pik3cb*^*+/+*^), *n* = 7 (*Pik3cb*^*D931A/D931A*^), *p < 0.05, (two-sided Student’s t-test (**A**) *p* = 0.0257, (**B**) *p* = 0.0113, (**c**) *p* = 0.0131, (**d**) *p* = 0.3128, (**e**) *p* = 0.7943) (**f**) Dot plot analysis of *Pik3ca*, *Pik3cb*, *Pik3cg*, *Pik3cd*, *Slc38a1*, *Slc38a2* and *Slc38a4* from single nuclei RNA-seq data of E9.5-E14.5 mouse trophoblasts from Marsh *et al.* 2020 [38]. Dot plot shows the average expression and percent of nuclei expressing each gene

We therefore assessed the expression of the amino acid transporters *Slc38a1*, *Slc38a2*, *Slc38a4* (encoding SNAT1, SNAT2 and SNAT4, respectively) and glucose transporters *Slc2a1* and *Slc2a3* in the E13.5 mutant placenta compared to controls. The system A amino acid transporter *Slc38a1*, *Slc38a2*, *Slc38a4* mRNA levels were all reduced in *Pik3cb*^*D931A/D931A*^ placentas (Fig. 5a-c, S5a-c). No robust changes in glucose transporter *Slc2a1* and *Slc2a3* mRNA levels were observed between genotypes (Fig. 5d-e, S5d-e; note a subtle but significant increase in *Slc2a3* mRNA levels was observed in female, but not male *Pik3cb*^*D931A/D931A*^ placentas).

The system A amino acid transporters are Na^+^-dependent and mediate the transfer of neutral amino acids to the embryo. Reduced system A amino acid transporter activity is well established in placentas of human newborns that are small for their gestational age [48] and with intrauterine growth restriction [49, 50]. Animal models have shown reduced system A mRNA expression and activity induces fetal growth restriction [21, 22, 51–55]. Therefore, these data suggest a defect in system A amino acid transporter expression may contribute to *Pik3cb*^*D931A/D931A*^ embryonic growth restriction and lethality.

We next assessed expression of the class I PI3K catalytic subunits and system A amino acid transporters in E9.5-E14.5 mouse placenta using the Marsh *et al.* 2020 single nuclei RNAseq dataset [38]. Both *Pik3ca* and *Pik3cb* were found to be expressed in the placenta, with a broader distribution of the latter. *Pik3ca* and *Pik3cb* showed a differential enrichment in placental cell types (Fig. 5f, S5f), with the highest *Pik3ca* levels in the maternally derived decidual stroma (Fig. S5f). Notably, *Pik3cb* expression extensively overlapped with the trophoblast subpopulations expressing *Slc38a1*, *Slc38a2*, and to a lesser extent, *Slc38a4* (Fig. 5f). Although the highest *Slc38a2* levels were observed in endothelial cells (Fig. 5f). The highest *Pik3cb* expression (in terms of number of cells and levels) was found in syncytiotrophoblast layer I (which mediates nutrient transport) and in spongiotrophoblasts/spongiotrophoblast precursors (forming the junctional zone) (Fig. 5f); cell types that were impacted in *Pik3cb*^*D931A/D931A*^ placentas (see Figs. 4 and 5a, b and c). *Pik3cg* and *Pik3cd* were expressed in very few cells and, although moderate, *Pik3cd* was detected in sinusoidal trophoblast giant cells and blood cells, consistent with its known predominant leukocyte distribution (Fig. 5f, S5f).

### Repression of AKT signalling in *Pik3b*^*D931A/D931A*^ placentas

**Fig. 6:**
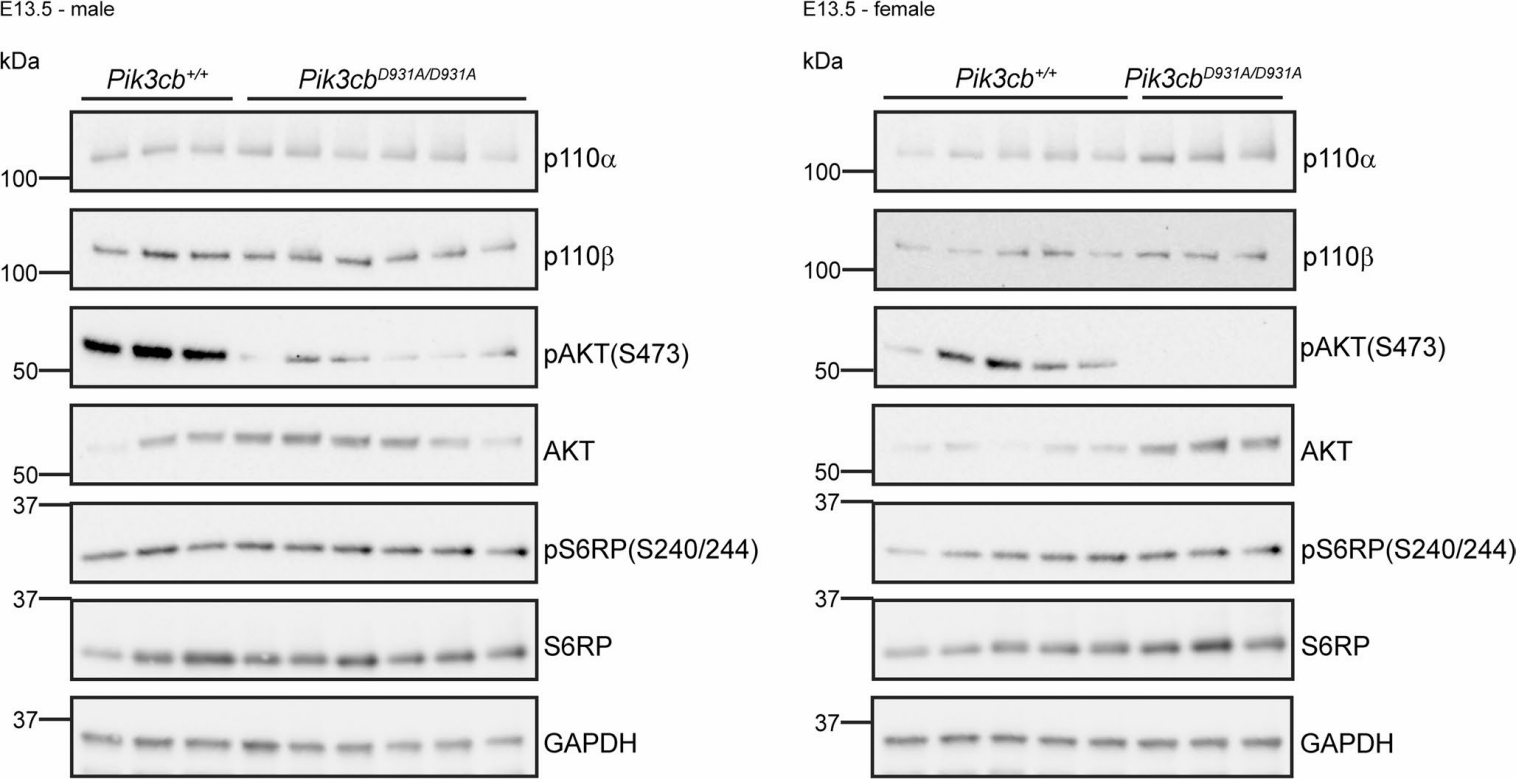
PI3Kβ inactivation downregulates AKT signalling in the placenta. E13.5 *Pik3cb*^*+/+*^ and *Pik3cb*^*D931A/D931A*^ placentas were lysed and immunoblotted using p110α, p110β, pAKT(S473), AKT, pS6RP(S240/244), S6RP or GAPDH antibodies. Each lane represents lysates from an individual mouse placenta

We next examined the signalling downstream of PI3Kβ in the placenta, as downregulation of PI3K effector activation has been previously reported in models of undernutrition and intrauterine growth-restricted placentas, in some cases, associated with reduced system A amino acid transporter expression [21, 22, 33, 56]. At E13.5, expression of the WT and kinase-dead p110β catalytic subunits and the other ubiquitously expressed class I PI3K isoform p110α were largely unchanged between genotypes (Fig. 6). Although there was some variation in the levels of total AKT between mice, phosphorylated and active pAKT(S473) was consistently reduced in both male and female *Pik3cb*^*D931A/D931A*^ placentas relative to sex-matched controls (Fig. 6). However, the activation of mTORC1 effector pS6RP(S2040/244) was unchanged by PI3Kβ inactivation (Fig. 6), likely because many upstream stimuli independent of PI3Kβ also impinge on mTORC1.

## Discussion

Of the class IA PI3K isoforms, the signalling input into PI3Kβ is the most complex given that this enzyme integrates signals from GPCRs, RTKs and small GTPases [23–25]. At present, the *in vivo* functions played by PI3Kβ remain incompletely understood, but appear to be overall less critical than those of PI3Kα or PI3Kδ under normal adult physiological conditions.

To try gain more insight into the functions of PI3Kβ relevant to both development and adult homeostasis, we performed an in-depth characterisation of the embryonic phenotype of p110β kinase-dead mice (*Pik3cb*^*D931A/D931A*^). The mechanisms underlying the reported partial but substantial embryonic lethality of these mice remains unclear. Here we show the majority of E10.5 and E13.5 *Pik3cb*^*D931A/D931A*^ embryos are largely normal, but growth-restricted. The *Pik3cb*^*D931A/D931A*^ placenta is small with a reduction in the endocrine and energy storing junctional zone, and a downregulation of the genes encoding the system A amino acid transporters, which are essential for nutrient transport to the embryo. The system A amino acid transporters SNAT1, SNAT2 and SNAT4 (encoded by *Slc38a1*, *Slc38a2* and *Slc38a4*, respectively) are expressed by the syncytiotrophoblast in the labyrinthine zone and transfer neutral amino acids to the embryo. Their expression and activity are reduced in placentas of human pregnancies with fetuses small for their gestational age and exhibiting intrauterine growth restriction [48–50, 57]. Animal models have also shown that reduced expression or activity of system A amino acid transporters causes intrauterine growth restriction [21, 22, 51–55]. Although a recent study also showed that reduced placental *Slc38a2* at late gestation may be a consequence of small embryo size [58]. Given our observations of reduced *Slc38a1*, *Slc38a2* and *Slc38a4* earlier in gestation and the previous literature, we propose that downregulation of these transporters in the *Pik3cb*^*D931A/D931A*^ placenta underlies the embryonic growth restriction and partial lethality. Interestingly, in some models with small placentas, system A amino acid transporters are *upregulated* as a compensatory mechanism to maintain embryonic growth in the presence other perturbations [4, 33, 59, 60]. Therefore, the observation of *reduced* system A amino acid transporter expression in *Pik3cb*^*D931A/D931A*^ placentas, suggests this is a primary defect driving the observed phenotype.

The precise mechanisms by which loss of p110β kinase activity reduces *Slc38a1*, *Slc38a2* and *Slc38a4* expression in the syncytiotrophoblasts, including the upstream activators of PI3Kβ signalling in this cell type, are incompletely understood, in part due to the highly dynamic nature and the challenges of experimentally manipulating the placenta. However, it is likely that the observed phenotype upon PI3Kβ inactivation is through the downregulation of placental AKT signalling, given AKT phosphorylation and activation was reduced in *Pik3cb*^*D931A/D931A*^ placentas. Repression of AKT is also reported in several intrauterine growth restriction placental models with reduced system A amino acid transporter expression [21, 22, 33, 56]. Furthermore, *Akt1* deletion reduces placental mass and embryonic growth, but system A amino acid transporter expression has not been assessed in this context [19]. Downstream mTORC1 signalling was unperturbed in the *Pik3cb*^*D931A/D931A*^ placentas, likely due to AKT-independent mTORC1 activation. However, mTORC1 has been shown to predominantly regulate the translocation of SNAT proteins to the plasma membrane, rather than promote their transcription [61, 62]. Together, these data argue for an AKT-dependent, mTORC1-independent, down-regulation of system A amino acid transporter expression as the likely mechanism in *Pik3cb*^*D931A/D931A*^ placentas. Consistently, we observed no gross histological changes in the labyrinthine zone, nor disruption of syncytiotrophoblast lineage marker expression in *Pik3cb*^*D931A/D931A*^ placentas, suggesting that the downregulation of the system A amino acid transporters is an altered signalling event in this cell type, rather than a consequence of abnormal syncytiotrophoblast progenitor specification or differentiation.

It is interesting to note that the major histological defect observed in *Pik3cb*^*D931A/D931A*^ placentas is a reduction in the junctional zone, consistent with the reduced expression of the markers for junctional zone spongiotrophoblast and giant cell lineages, but the ratio of cell types within this region was unperturbed. We quantified the junctional zone cell type composition using point counting of H&E-stained placentas, which is a robust technique given the distinctive cell type morphologies and lack of specific antibodies to markers defining these populations for immunohistochemistry. In WT mice, naturally small placentas also exhibit a specific reduction in the junctional zone compared to large placentas within the same litter [4]. The junctional zone is also specifically compromised at E16.5 following under-nutrition of pregnant mice [60]. Given the junctional zone stores energy in glycogen cells and produces growth factors and hormones [42], it is hypothesised that the reduced junctional zone proportion in small placentas is a compensatory mechanism to preferentially partition nutrients to the embryo at the expense of storage and utilisation by the placenta [4]. This hypothesis may also apply to the *Pik3cb*^*D931A/D931A*^ placenta whereby the primary defect in amino acid transport in the labyrinthine zone leads to a compensatory diversion of nutrients to the embryo, at the expense of the junctional zone. A loss of junctional zone hormone production may also further contribute to reduced embryo growth [63]. However, we cannot exclude the possibility that p110β inactivation also disrupts the balance of trophoblasts progenitor differentiation into labyrinthine and junctional zone lineages which would be interesting to assess in future studies.

Although we have identified a mechanism for growth restriction at E10.5 to E13.5, a proportion of *Pik3cb*^*D931A/D931A*^ embryos die prior to E10.5 and a very small proportion of surviving embryos exhibit more severe patterning defects. Given that definitive placental cell type differentiation is only initiated at around E8.5, maternal blood enters the labyrinthine zone by E9.5 and haemotrophic nutrition is initiated after E10 [64], non-placental processes regulated by PI3Kβ likely contribute to the defects in these embryos. Furthermore, we previously reported that on a C57BL/6 background only 1% of pups from heterozygous inter-crosses surviving to weaning were *Pik3cb*^*D931A/D931A*^ [27], compared to 16.6% observed here at E13.5 on the same background. Therefore, *Pik3cb*^*D931A/D931A*^ placental insufficiency and growth restriction may progress and manifest as lethality in later stage fetuses and/or neonates or additional mechanism/s may also contribute. Furthermore, feto-placental development can be influenced by embryo sex [65]. We examined embryos with sexes combined and stratified by sex, observing consistent patterns in overall phenotype, however, the stratified analysis was potentially underpowered to detect subtle sex-specific differences and could be strengthened using a larger sample size in future studies. Nevertheless, we observed some sex-specific placental adaptions to PI3Kβ inactivation. Junctional zone spongiotrophoblasts were reduced and giant cells increased specifically in male placentas, whereas the glucose transporter *Slc2a3* mRNA level was specifical increased in female placentas. Similarly, *Slc2a3* mRNA levels have been previous shown to be specifically upregulated in female placentas in a murine lipopolysaccharide-induced intrauterine growth restriction model [66]. The mechanisms driving sex-specific placental adaption are only beginning to emerge and have been suggested to involve differential metabolic, epigenetic, transcriptional and hormonal impacts [66–69].

While it is not possible to directly compare the placental phenotype of *Pik3cb*^*D931A/D931A*^ with *Pik3ca*^*D933A/D933A*^ embryos due to lethality of the latter at around E10.5 [8], *Pik3ca*^*D933A/+*^ embryos do exhibit a placental phenotype and growth restriction [18]. Unlike *Pik3cb*^*D931A/D931A*^ placentas with reduced expression of system A amino acid transporters and histological disruption of the junctional zone, the labyrinthine zone and surface area for nutrient exchange are reduced in *Pik3ca*^*D931A/+*^ placentas. While p110α and p110β are considered to be ubiquitously expressed, including in the placenta, we show here these isoforms are enriched in distinct placental cell types and are clearly non-redundant in placental function with p110α perhaps more important for labyrinthine zone histological architecture and p110β regulating nutrient exchange.

PI3Kα seems to be the predominant isoform in many adult contexts, such as in insulin and glucose homeostasis and IGF-1 signalling, with PI3Kα preferentially active in IRS1 and IRS2 complexes following insulin or IGF stimulation [16]. This is unlike PI3Kβ, given that no defects are observed in insulin- or IGF-stimulated AKT activation in cells derived from *Pik3cb* homozygous knockout [35] or *Pik3cb* kinase-dead mice (shown here), or cells treated with PI3Kβ inhibitors [35]. Although p110β kinase-dead mice (*Pik3cb*^*K805R/K805R*^) mice exhibit mild insulin resistance from 6 months of age [28], it is suggested that PI3Kβ may only set a basal level of PI3K activity allowing for stimulus-induced PI3Kα activity to overcome the threshold for downstream pathway activation [70]. Consistently, *PIK3CB* is not frequently mutated in cancer, but appears to play an important role in tumours with loss of the PIP_3_ phosphatase *PTEN* in some but not all contexts [71–74]. p110β kinase-dead mice that survive development are relatively healthy and, although they remain growth-restricted up to 4 weeks of age, by 12 weeks onward there is no difference in their mass compared to WT controls [27]. PI3Kβ inhibitors have been developed for use in humans and tested in males and females for oncology and as an anti-thrombotic with favourable tolerability profiles in these contexts compared to inhibitors of other PI3K isoforms [30–32], although they have not been tested in pregnant females. These observations indicate that PI3Kβ activity is not essential for normal adult homeostasis. Here we identified that PI3Kβ kinase activity promotes the expression of the system A amino acid transporters. *Slc38a1* and *Scl38a2* are variably, but widely expressed in adult tissues and *Scl38a4* is expressed in the liver [53]. Consistent with the essential function of PI3Kβ in the embryo, but not the adult, inhibition of system A amino acid transporters induces fetal growth restriction, but has no overt detrimental impact on pregnant adult female rats [55]. Furthermore, the SNAT protein knockout mouse models that survive the embryonic/neonatal period [53, 75, 76], seem to display profound phenotypes mainly under stressed conditions [76]. Interestingly, cancer patients treated with the PI3Kβ-selective inhibitor GSK2636771 display hypokalemia and hypocalcemia without overall kidney dysfunction [77, 78]. Such adverse effects have not reported for any inhibitor of other PI3K isoforms, and it is tempting to speculate that p110β might also control the expression of other Solute Carrier (SLC) superfamily family members, such as those involved in the transport of phosphate and calcium in the kidney and other tissues.

Collectively, using the sensitive biological system of embryonic and placental development, we have uncovered a novel function for PI3Kβ in regulation of system A amino acid transporter expression. Amino acid transfer to the embryo via the placenta is critical for embryonic growth, therefore we propose its downregulation underlies *Pik3cb*^*D931A/D931A*^ placental insufficiency, embryonic growth restriction and partial lethality. However, PI3Kβ and the system A amino acid transporters appear to be less critical in the adult, other than under stress and for pregnancy, which may explain the favourable tolerability of PI3Kβ inhibitors tested in non-pregnant adults.

## Supplementary Information

Below is the link to the electronic supplementary material.

Supplementary Material 1 (PDF 1.10 MB)

## Data Availability

The single nuclei RNAseq dataset analysed during the current study is available from figshare, https://figshare.com/projects/Single_nuclei_RNA-seq_of_mouse_placental_labyrinth_development/92354. Other datasets generated during the current study are not publicly available due to the lack of dedicated repositories for the data types but are available from the corresponding authors on request.

## Abbreviations

AWERB: animal welfare and ethical review body
E: embryonic day
FFPE: formalin-fixed, paraffin-embedded
GPCR: G protein-coupled receptor
H&E: haematoxylin and eosin
IGF-1: insulin like growth factor-1
IGF1R: insulin-like growth factor 1 receptor
MEF: mouse embryonic fibroblast
PI3K: phosphoinositide 3-kinase
RTK: receptor tyrosine kinases
SDS: sodium dodecyl sulphate
v/v: volume/volume
WT: wild-type
w/v: weight/volume
