# Supplementary material for: Novel role for PI3Kβ in placental function through regulation of system A amino acid transporter expression, associated with embryonic lethality
Source: Cell Mol Life Sci. 2025 Nov 19;82(1):413. doi: 10.1007/s00018-025-05937-w (PMC12630484; doi:10.1007/s00018-025-05937-w)
Supplement: Supplementary file 1 — Supplementary Material 1 (PDF 1.10 MB) [file 18_2025_5937_MOESM1_ESM.pdf]

Novel role for PI3Kβ in placental function through regulation of system A amino acid transporter expression, associated with embryonic lethality

Cellular and Molecular Life Sciences

Sarah E. Conduit[1,*], Cindy X. W. Zhang[2], Wayne Pearce[1], Julie Guillermet-Guibert[3,4] Amanda N. Sferruzzi-Perri[2], Bart Vanhaesebroeck[1,*]

[1]Cell Signalling, UCL Cancer Institute, University College London, London, UK.
[2]Department of Physiology, Development, and Neuroscience, Loke Centre for Trophoblast Research, University of Cambridge, Cambridge, UK.
[3]Labex Toucan, Toulouse, France.
[4]Université de Toulouse, Inserm, CNRS, CRCT, Centre de Recherches en Cancérologie de Toulouse, Toulouse, France.

*Correspondence: Sarah E. Conduit (s.conduit@ucl.ac.uk) or Bart Vanhaesebroeck (bart.vanh@ucl.ac.uk)

Supplementary Figures

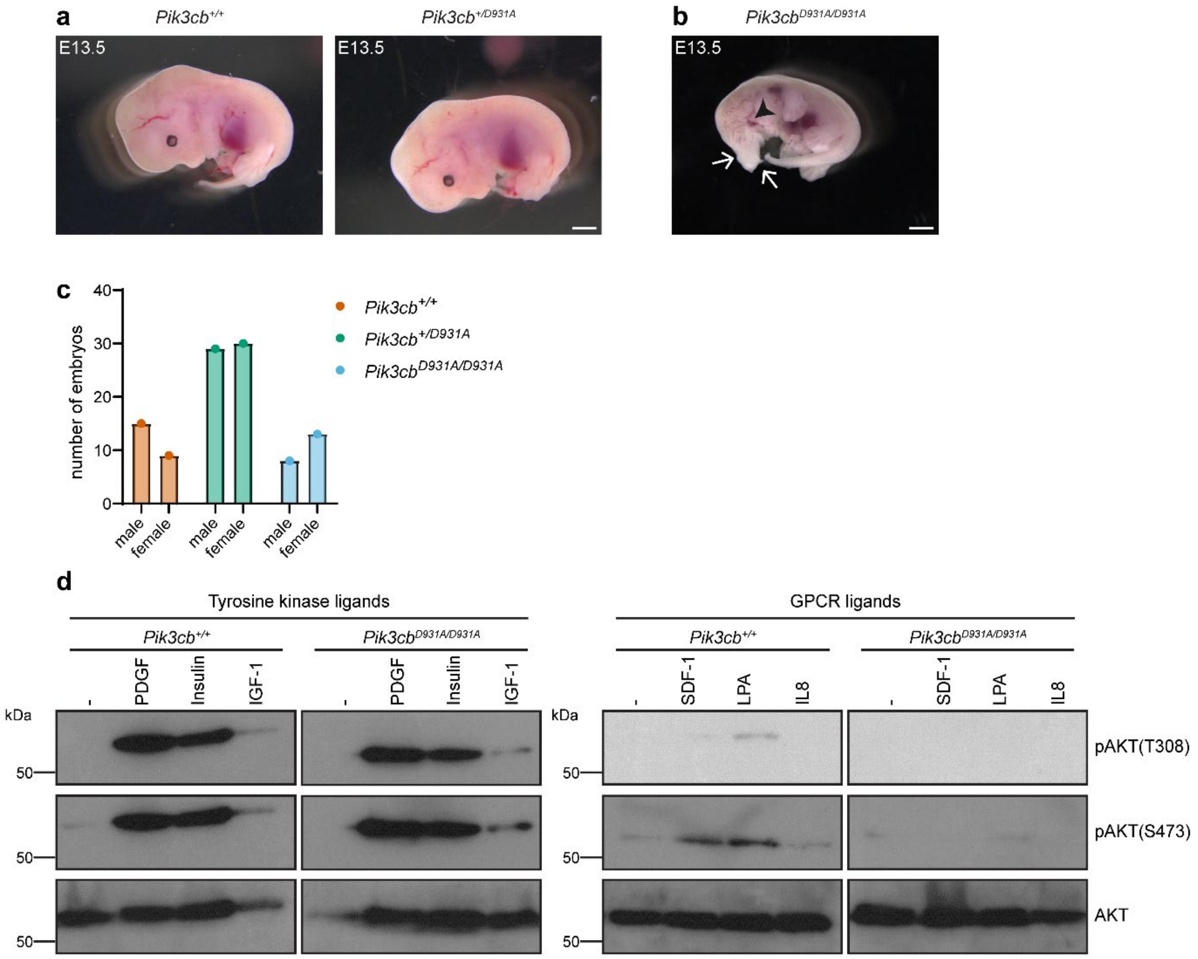

Fig. S1

PI3Kβ inactivation impacts the signalling response to GPCR but not RTK ligands

a. Whole mount images of E13.5 *Pik3cb*$^{+/+}$ and *Pik3cb*$^{+/D931A}$ embryos, scale bar: 1 mm, representative of n=20 (*Pik3cb*$^{+/+}$), n=31 (*Pik3cb*$^{+/D931A}$) embryos

b. Whole mount images of E13.5 *Pik3cb*$^{D931A/D931A}$ embryos, scale bar: 1 mm, arrow indicates abnormal craniofacial region and arrowhead indicates anophthalmia, representative n=2 embryos with severe patterning defects from n=28 *Pik3cb*$^{D931A/D931A}$ embryos

c. Sex ratio of male and female *Pik3cb*$^{+/+}$ and *Pik3cb*$^{+/D931A}$, *Pik3cb*$^{D931A/D931A}$ embryos at E13.5

d. *Pik3cb*$^{+/+}$ and *Pik3cb*$^{D931A/D931A}$ MEFs were starved and stimulated for 10 min with PDGF, Insulin, IGF-1, SDF-1, LPA or IL8. Total cell lysates were immunoblotted with pAKT(T308), pAKT(S473) or total AKT antibodies. A representative immunoblot from n=2 independent experiments is shown. Membranes were probed with pAKT(T308) antibodies, stripped and re-probed with pAKT(S473) antibodies

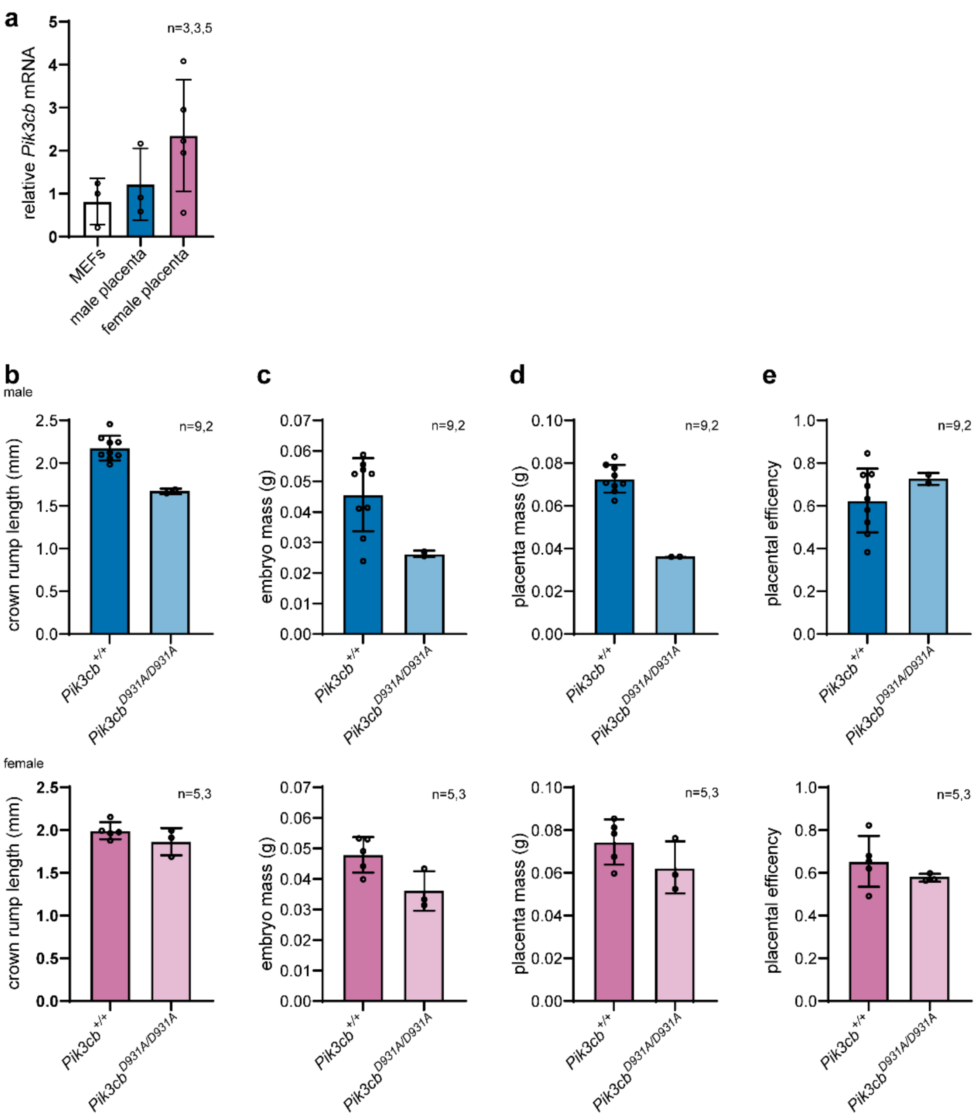

Fig. S2

Trend for reduced feto-placental growth E10.5 PI3Kβ kinase-dead mice stratified by sex

a. MEFs and E13.5 placentas were lysed and *Pik3cb* mRNA levels quantified by qRT-PCR relative to *Actb*. Bars represent mean ± SD, n=3 MEFs, n=3 (male), n=5 (female) placentas

b-d. E10.5 male or female $Pik3cb^{+/+}$ and $Pik3cb^{D931A/D931A}$ (**b**) embryo crown-rump length, (**c**) embryo mass, (**d**) placental mass and (**e**) placental efficiency. Bars represent mean ± SD, n=9 (male $Pik3cb^{+/+}$), n=2 (male $Pik3cb^{D931A/D931A}$), n=5 (female $Pik3cb^{+/+}$), n=3 (female $Pik3cb^{D931A/D931A}$) embryos (single measures parametric analysis, with litter as a block factor (**b** female) p=0.1814, (**c** female) p=0.0796, (**d** female) p=0.5473, (**e** female) p=0.0579)

Conduit *et al.*

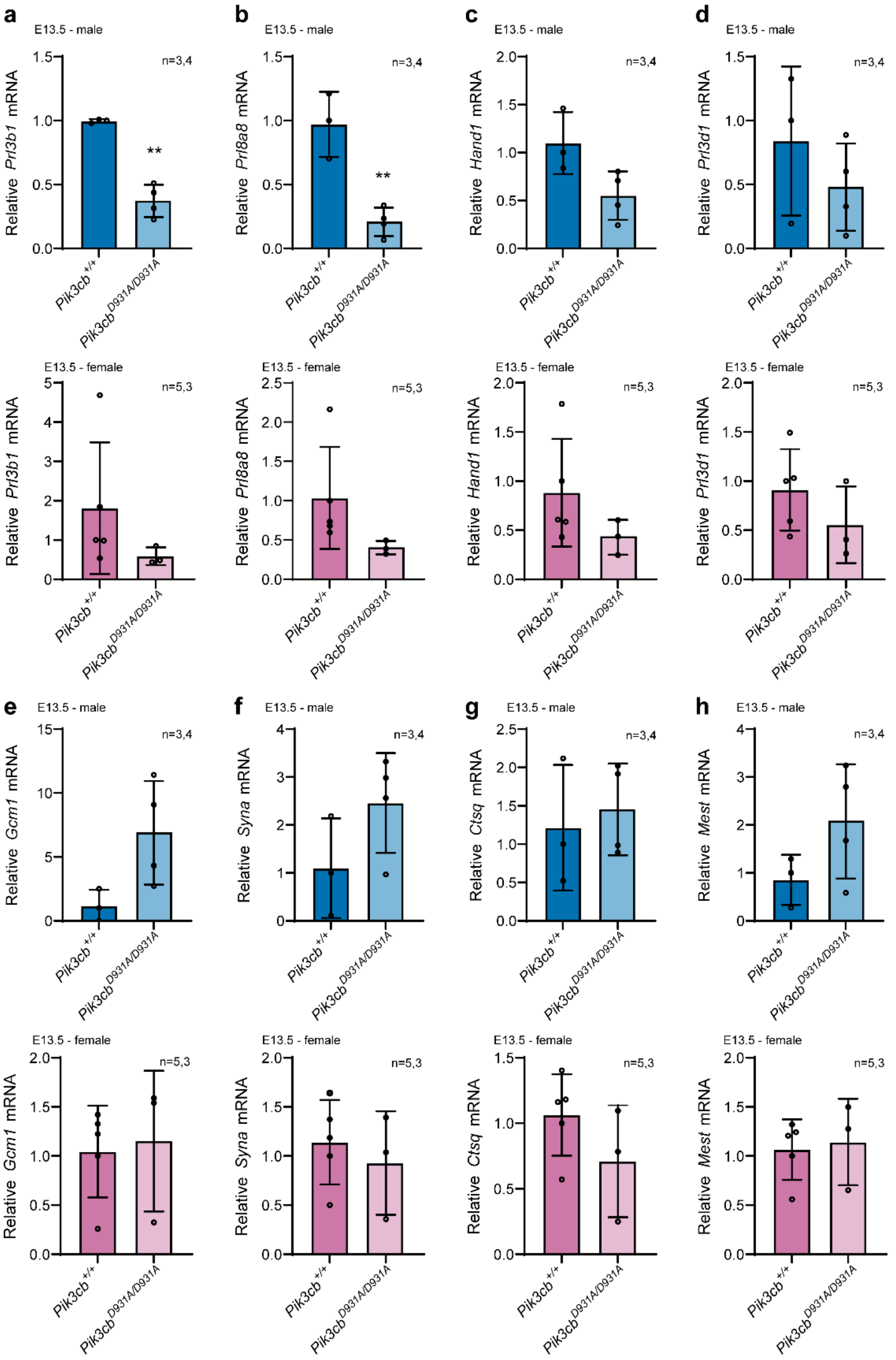

Fig. S3

Junctional zone linage marker expression is reduced in PI3Kβ kinase-dead placentas **a-h.** E13.5 *Pik3cb*$^{+/+}$ and *Pik3cb*$^{D931A/D931A}$ placentas were lysed and (**a**) *Prl3b1*, (**b**) *Prl8a8*, (**c**) *Hand1*, (**d**) *Prl3d1*, (**e**) *Gcm1*, (**f**) *Syna*, (**g**) *Ctsq* or (**h**) *Mest* mRNA levels quantified by qRT-PCR relative to *Actb*. Bars represent mean ± SD, n=3 (male *Pik3cb*$^{+/+}$), n=4 (male *Pik3cb*$^{D931A/D931A}$), n=5 (female *Pik3cb*$^{+/+}$), n=3 (female *Pik3cb*$^{D931A/D931A}$) placentas, **$p < 0.01$ (two-sided Student's t-test (**a**) male p=0.0019,

female p=0.1801, (**b**) male p=0.0028, female p=0.0945, (**c**) male p=0.0522, female p=0.2243, (**d**) male p=0.3456, female p=0.2775, (**e**) male p=0.0685, female p=0.8065, (**f**) male p=0.1479, female p=0.5568, (**g**) male p=0.6706, female p=0.2199, (**h**) male p=0.1648, female p=0.7782)

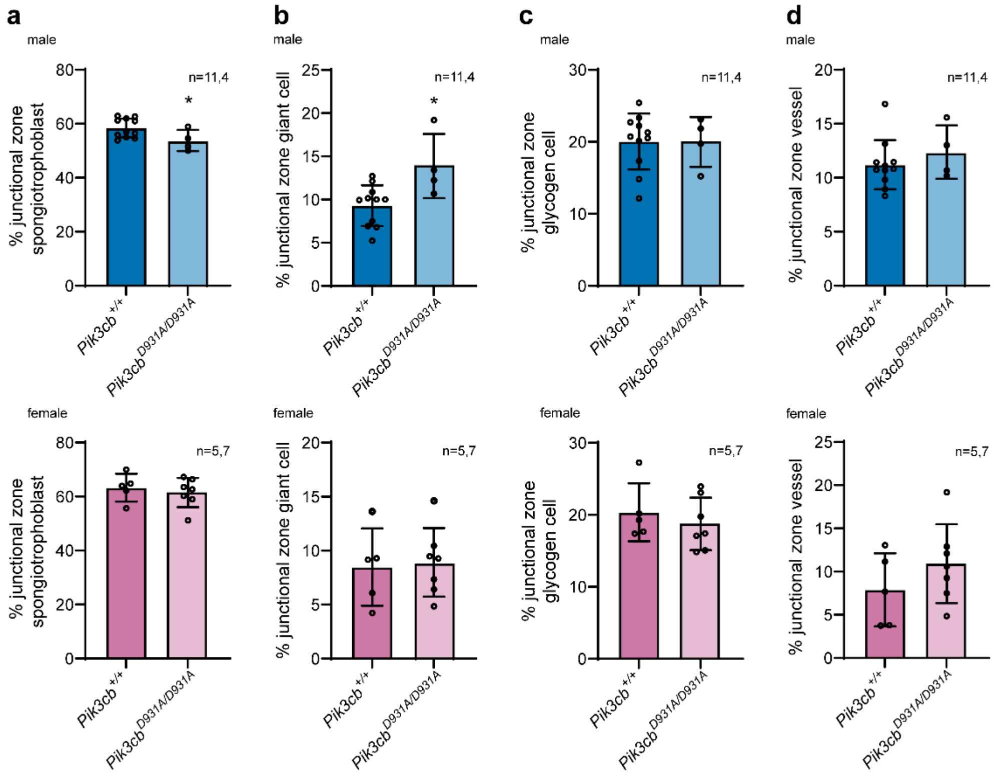

Fig. S4

PI3Kβ inactivation has little effect on the junctional zone cell type distribution

a-d. E13.5 *Pik3cb*$^{+/+}$ and *Pik3cb*$^{D931A/D931A}$ placenta sections were stained with H&E. The percentage of the junctional zone occupied by the (**a**) spongiotrophoblast, (**b**) giant cells, (**c**) glycogen cells or (**d**) vessels was quantified. Bars indicate mean ± SD, n=11 (male *Pik3cb*$^{+/+}$), n=4 (male *Pik3cb*$^{D931A/D931A}$), n=5 (female *Pik3cb*$^{+/+}$), n=7 (female *Pik3cb*$^{D931A/D931A}$) placentas of each genotype (two-sided Student's t-test (**a**) male p=0.0473, female p=0.5671, (**b**) male p=0.2081, female p=0.0113, (**c**) male p=0.9720, female p=0.4848, (**d**) male p=0.4068, female p=0.2709)

Conduit *et al.*

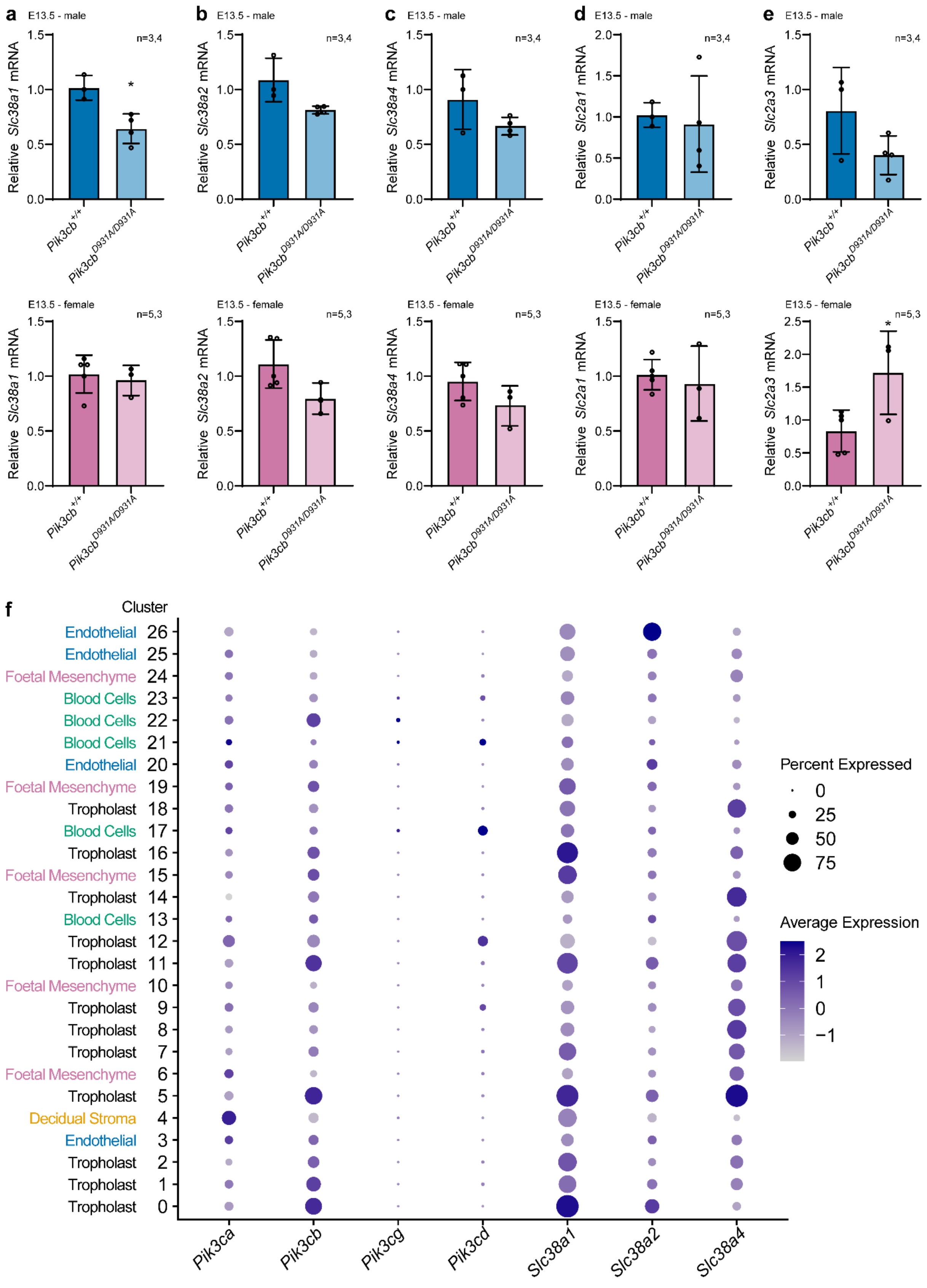

Fig. S5

Trend for reduced System A amino acid transporter expression in PI3Kβ kinase-dead placentas stratified by sex

a-h. E13.5 *Pik3cb*$^{+/+}$ and *Pik3cb*$^{D931A/D931A}$ placentas were lysed and (**a**) *Slc38a1*, (**b**) *Slc38a2*, (**c**) *Slc38a4*, (**d**) *Slc2a1*, (**e**) *Slc2a3* mRNA levels quantified by qRT-PCR relative to *Actb*. Bars represent mean ± SD, n=3 (male *Pik3cb*$^{+/+}$), n=4 (male *Pik3cb*$^{D931A/D931A}$), n=5 (female *Pik3cb*$^{+/+}$), n=3 (female *Pik3cb*$^{D931A/D931A}$) placentas, *$p < 0.05$ (two-sided Student's t-test (**a**) male p=0.0119, female p=0.6383, (**b**) male p=0.1373, female p=0.0710, (**c**) male p=0.1400, female p=0.1352, (**d**) male p=0.7691, female p=0.6449, (**e**) male p=0.1201, female p=0.0350)

(**f**) Dot plot analysis of *Pik3ca*, *Pik3cb*, *Pik3cg*, *Pik3cd*, *Slc38a1*, *Slc38a2* and *Slc38a4* from single nuclei RNA-seq data of E9.5-E14.5 mouse placenta from Marsh *et al.* 2020 [37]. Dot plot shows the average expression and percent of nuclei expressing each gene. Some cell types consist of several subpopulations which fall into distinct clusters as indicated on the y-axis
